# Sport climbing competence is influenced by training frequency, experience, self-efficacy, flow, and emotional intelligence

**DOI:** 10.3389/fpsyg.2025.1518495

**Published:** 2025-06-06

**Authors:** Marina Turchetto, Valentina Tomaselli, Francesca Giorgi, Silvia Leone, Irene Leo

**Affiliations:** Department of Developmental and Social Psychology, University of Padua, Padua, Italy

**Keywords:** embodied cognition, emotional intelligence, self-esteem, flow, mindfulness, climbing, self-efficacy

## Abstract

Sport climbing requires the integration of physical and cognitive abilities. The Embodied Cognition (EC) theoretical framework is increasingly applied in recreational sport research, as it emphasizes the influence of bodily experience on cognition functioning. This exploratory study investigated the relationship between competence in sport climbing and several psychological factors, including both stable traits (e.g., self-esteem, trait emotional intelligence) and trainable capacities (e.g., mindfulness, self-efficacy, flow), as well as climbing experience and training frequency. A sample of 365 climbers (40% female, M = 32.11 years) completed questionnaires. Correlation analysis showed positive associations between climbing competence and training frequency, years of practice, mindfulness, and climbing self-efficacy. A structural equation model (SEM) revealed that climbing confidence was the strongest psychological predictor of competence, while mindfulness unexpectedly emerged as a negative predictor. Trait emotional intelligence was positively associated with mindfulness but not directly with competence. A bidirectional relationship between mindfulness and competence was observed, suggesting a complex interaction. These findings highlight the interplay between psychological dispositions and sport-specific skills in climbing performance.

## Introduction

The Embodied Cognition (EC) approach has significantly changed the way researchers conceptualize sensorimotor experience. From this perspective, the body and its sensorimotor patterns play a crucial role in all cognitive processes (Wellsby and Pexman, 2014; Vallet et al., 2016). Mental activity is shaped by the ongoing interaction between the body and its environment (Leo et al., 2022). In this light, EC offers a valuable lens for understanding athletic performance as a dynamic interaction between mind and body (Raab and Araújo, 2019), particularly in activities such as climbing, which require an intricate balance of physical and cognitive skills (Stephan et al., 2011; Wickens et al., 2018).

Sport climbing is a distinct discipline within the broader domain of climbing sports, which also includes traditional climbing and ice climbing. According to the Italian Federation of Sport Climbing (Federazione Arrampicata Sportiva Italiana [FASI], 2025), sport climbing involves climbing for competitive, recreational, or educational purposes on either artificial or natural walls along predefined, equipped routes. Artificial aids for progression are not permitted. There are three types of sport climbing: lead climbing, speed climbing, and bouldering, which are now formally recognized at the international level and were included in the Olympic Games beginning with Tokyo 2021. Lead climbing involves ascending a route using a belayed dynamic rope, either indoors on artificial walls or outdoors on natural rock. Speed climbing uses a standardized route equipped with a timing system and typically employs top-rope belaying, often with automatic belayers. Bouldering consists of short, high-intensity climbs performed without ropes but with crash mats for protection.

Despite the formalization and growing popularity of sport climbing, much of the psychological literature has addressed “rock climbing” in general, often without clearly distinguishing between its subdisciplines. This terminological ambiguity can obscure the specificity of findings and limit their theoretical and pratical utility. Moreover, climbing has frequently been studied through a therapeutic lens, with an emphasis on its benefits for mental health rather than as a performance based acitivity. Research has shown that climbing can reduce anxiety (Aras and Ewert, 2016), alleviate depression (Zieliński et al., 2018), and improve self-esteem and self-confidence (Bahaeloo-Horeh and Assari, 2008; Steimer and Weissert, 2017). While these findings remain important, a growing body of research highlights the role of psychological variables in predicting climbing performance (Mangan et al., 2024).

Classical psychological factors such as personality (Ionel et al., 2023; Steinmetz et al., 2022) and self-efficacy (Llewellyn and Sanchez, 2008; Saul et al., 2019) have been widely examined. Self-efficacy refers to an individual’s belief in their ability to organize and execute the actions required to achieve specific outcomes (Bandura, 1997). It is a strong predictor of the difficulty, frequency, and risk level of climbing activities, with more self-efficacious individuals tending to pursue more challenging goals (Bandura, 1997). Self-efficacy should not be confused with self-esteem, which pertains to an individual’s general evaluation of their own self-worth (Rosenberg, 1965).

Additionally, flow experience appears to be a relevant factor in rock climbing performance (Mangan et al., 2024), especially in outdoor settings (Boudreau et al., 2022). Flow is defined as a state of deep concentration and immersion, characterized by a balance between skill and challenge (Csikszentmihalyi, 1990; 2008; Csikszentmihalyi and LeFevre, 1989). More recent studies view flow as a dynamic psychological process rather than a fixed state (Šimleša et al., 2018). Boudreau et al. (2022) found that rock climbers reported experiencing flow in at least one of their climbs in 72% of outdoor sessions. Interestingly, flow states often preceded the phase of route exploration—a critical component of successful climbing (Rucińska, 2021). Nonetheless, the link between flow and sport climbing performance warrants further investigation.

Other psychological variables have received comparatively less attention. For example, Garrido-Palomino and España-Romero (2019) reported a relationship between Emotional Intelligence (EI) and sport climbing performance. EI involves the capacity to pursue long-term goals while employing adaptive emotional regulation strategies (Laborde et al., 2012; Laborde et al., 2014a,b). Variations in EI may differentiate sport climbers across skill levels, suggesting its potential role in performance (Garrido-Palomino and España-Romero, 2019). Although EI’s importance in sports performance is well documented (Laborde et al., 2011; Laborde et al., 2015), further research is needed to clarify its specific role in sport climbing.

Given the stressful nature of sport rock climbing, athletes must maintain focus on the present moment and manage anxiety and fear of falling (Aras and Akalan, 2014). Fear of falling in sport climbing is considered as an element of psychological stress (Garrido-Palomino and España-Romero, 2023) that may impair the performance of lower grade and intermediate climbers, but not in advanced and élite ones (Gajdošík et al., 2020). Mindfulness may enhance sport climbing ability by reducing fear (Ilgner, 2003), improving breathing control, and supporting performance visualization. Mindfulness is defined as “paying attention in a particular way: on purpose, in the present moment, and non-judgmentally” (Kabat-Zinn, 1994). It is inherently embedded in climbing activities (Wheatley, 2023), promoting mental skills necessary to stay focused under pressure (Young and Knight, 2014), manage emotional reactions, and confront fear (Steca et al., 2018). Notably, Wheatley (2023) found that young adults engaged in rock climbing exhibited higher mindfulness levels compared to peers involved in other physical activities. These findings suggest a bidirectional relationship: rock climbing may improve mindfulness, and mindfulness may, in turn, enhance rock climbing performance. However, no studies to date have directly explored this two-way interaction.

Guided by the EC framework, this study focuses on the psychological underpinnings of competence in sport climbing. In contrast to prior research that often conflated distinct climbing disciplines, we specifically examined sport climbers. The psychological variables selected include a mix of enduring traits (e.g., self-esteem, trait emotional intelligence), acquires skills (e.g., self-efficacy), and trainable mental states or capacities (e.g., mindfulness, flow). This distinction reflects the complex nature of psychological functioning in sport contexts.

Based on previous findings, we hypothesized that competence in sport climbing would be positively associated with self-efficacy, flow experience, and self-esteem. Given the limited and mixed findings in the literature, we also explored the roles of trait emotional intelligence and mindfulness, without making strong directional predictions. We further expected training frequency and years of practice to contribute to higher competence, both directly and indirectly, through their influence on psychological functioning.

## Materials and methods

A total of 365 Caucasian individuals from Northern Italy participated in this exploratory study, including 135 females (40%) with an age range of 18–72 years (M = 32.11, SD ± 9.92). Over 60% were aged between 18 and 29. Approximately 33% reported 4–9 years of experience in sport climbing (M = 2.99, SD = 1.74), and trained around two times a week (M = 3.76, SD = 1.17). Based on Draper et al. (2011) classification, 46% self-identified at an intermediate level (grades 5b–6b), and 13% reported beginners-level competence (grades 4a–5a) (M = 2.43, SD = 0.94).

Almost all participants reported practicing both lead climbing and bouldering, the two most common sub-disciplines of sport climbing. They typically train in climbing gyms equipped with both indoor bouldering areas and lead walls, the latter often located just outside the gym facility. Recruitment was conducted through word of mouth and social media platforms. Individuals under the age of 18, those who had never practiced climbing, or those who had ceased practicing were excluded from participation. Data were collected between January and April 2021. All participants gave informed consent in accordance with the ethical standards of the Department of Psychology at the University of Padua, Italy.

### Measures

The questionnaire was administered online and consisted of six scales.

The Italian translation of the Mindful Attention Awareness Scale (MAAS) (Brown and Ryan, 2003) was used to assess mindfulness. The items are presented in reverse form, exploring how often individuals act automatically, without paying attention, or in a worried state. The scale includes 15 items rated on a 6-point Likert scale, ranging from 1 (“almost always”) to 6 (“almost never”); higher scores indicate a greater tendency toward mindfulness.

The Italian version of the Dispositional Flow Scale-2 (DFS-2) (Boffi et al., 2012; Jackson and Eklund, 2002) was used to measure individuals’ predisposition to experience flow. The scale comprises 36 items covering the nine dimensions of flow as proposed by Csikszentmihalyi (1990). Each subscale consists of four items rated on a 5-point Likert scale, where 1 corresponds to “never” and 5 to “always.” The flow dimensions include: challenge-skill balance (D1), action-awareness merging (D2), clear goals (D3), unambiguous feedback (D4), concentration on the task at hand (D5), sense of control (D6), loss of self-consciousness (D7), transformation of time (D8), and autotelic experience (D9).

Self-esteem was assessed using the Italian version of the Rosenberg Self-Esteem Questionnaire (RSEQ) (Prezza et al., 1997; Rosenberg, 1965), which consists of 10 items—five reflecting positive attributes and five capturing negative self-evaluations. Responses are given on a 4-point Likert scale ranging from 3 (“strongly agree”) to 0 (“strongly disagree”), resulting in a total score for self-esteem.

Emotional intelligence was measured using the Trait Emotional Intelligence Questionnaire—Short Form (TEIQue-SF) (Petrides, 2009), validated in Italian by Di Fabio and Palazzeschi (2011). The TEIQue-SF consists of 30 items rated on a 7-point Likert scale, where 1 indicates “strongly disagree” and 7 indicates “strongly agree.” It is a condensed version of the original TEIQue (Petrides and Furnham, 2001) and assesses global trait emotional intelligence (TEIQue Tot), along with four subscales: emotionality (TEIQue_EM), sociability (TEIQue_S), self-control (TEIQue_Sc), and well-being (TEIQue_W).

Self-efficacy was measured using the Climbing Self-Efficacy Scale (CSES) (Llewellyn and Sanchez, 2008), which was translated into Italian for this study. The scale includes 10 items aligned with Bandura (1997) theory of self-efficacy. Respondents rate their confidence in climbing-specific skills on a scale from 0% (“not at all confident”) to 100% (“extremely confident”). The total score ranges from 0 to 1000 and provides an indication of the individual’s belief in their ability to perform in the sport.

Participants were also asked to report their climbing competence level and training frequency for the year in which the questionnaire was completed. Climbing competence was defined by the highest climbing grade achieved that year, using the French grading system (from 3 to 9, with subgrades a, b, and c). Draper et al. (2011) scale was used to convert these grades into standardized ability levels.

### Data analysis

A descriptive analysis of the raw data, including means and standard deviations, was conducted using a Python program. The reliability of the measures was assessed through correlation analysis using the Spearman-Brown coefficient.

Subsequently, a Structural Equation Model (SEM) was constructed using the statistical software R. The model’s goodness of fit was evaluated using the Chi-Square test, where non-significant values indicate a good fit between the model and the data (Iacobucci, 2010).

In addition, several incremental goodness-of-fit indices were considered. The Standardized Root Mean Square Residual (SRMR) was evaluated, with values below 0.08 considered acceptable (Hu and Bentler, 1999). The Root Mean Square Error of Approximation (RMSEA) was also used, where values of 0.05 or lower indicate a good model fit (Browne and Cudeck, 1992). The Tucker-Lewis Index (TLI) and the Comparative Fit Index (CFI) were considered acceptable when values met or exceeded the threshold of 0.90 (Bentler, 1990; Tucker and Lewis, 1973).

Mindfulness, self-efficacy, self-esteem, flow, and emotional intelligence were included as psychological variables hypothesized to influence climbing competence both directly and indirectly. Years of practice and training frequency were also included in the model as additional variables, to more comprehensively capture the factors influencing performance.

### Preliminary analyses

Descriptive statistics and internal reliability coefficients for all the factors are provided in Table 1. The Cronbach’s alpha levels for each of the measures exceeded acceptable thresholds, indicating sufficient internal reliability for their inclusion in the analysis. Some subscales of the Teique scale exhibited slightly lower, yet still acceptable, internal consistency, specifically Teique EM (α = 0.68), Teique S (α = 0.66), and similarly, D2 of the flow scale also displayed low internal consistency (α = 0.68). Data related to participant groups categorized based on years of practice (YC), training frequency (TF), and level of expertise (LC) are represented in Table 2.

**TABLE 1:** Descriptive statistics and internal consistency (Cronbach’s α) for study variables.

Scales and subscales	M	SD	α
Mindfulness (MAAS)	3.99	0.83	0.88
Self-esteem (RSES)	1.98	0.58	0.88
Trat emotional intelligence factor (TEIQue Tot)	4.79	0.72	0.87
Emotionality (TEIQue_EM)	4.81	0.95	0.68
Self-control (TEIQue_Sc)	4.61	0.95	0.63
Wellbeing (TEIQue_W)	5.13	1.15	0.85
Sociability (TEIQue_S)	4.47	0.99	0.66
Dispositional flow total score (DFS tot)	3.39	0.49	0.92
Challenge-skills balance (D1)	3.41	0.63	0.79
Action-awareness merging (D2)	2.91	0.63	0.68
Clear goals (D3)	3.41	0.79	0.88
Unambiguous feedback (D4)	3.32	0.71	0.83
Concentrations on the task at hand (D5)	3.50	0.76	0.86
Sense of control (D6)	3.44	0.70	0.86
Loss of self-consciousness (D7)	3.23	0.99	0.87
Transformation of time (D8)	3.26	0.91	0.86
Autotelic experience (D9)	4.05	0.66	0.81
Climbing Confidence (CSES)	6.49	1.77	0.94

**TABLE 2:** Participant characteristics by climbing experience, training, and competence.

	n	%
**Years of climbing (YC)**		
YC beginner	57	15%
YC 1 year climbing	29	7%
YC 2 years climbing	44	12%
YC 3 years climbing	44	12%
YC 4-9 years climbing	112	33%
YC + 10 years climbing	79	21%
**Training frequency (TF)**		
TF 1 in a month	23	6%
TF 2/3 in a month	32	8%
TF 1 in a week	68	18%
TF 2 in a week	128	35%
TF 2 + in a week	114	31%
**Level of competence (LC) following draper scale**		
Beginners (4a-5a)	48	13%
Intermediate (5b-6b)	169	46%
Advanced (6b + -7a +)	101	27.7%
Experts (7b-7c +)	37	10%
Super experts (8a-8a +)	10	2.7%

Table 3 reports all the Spearman-Brown correlations between competence and years of practice, training frequency, and psychological factors. The data reveal positive and statistically significant correlations between competence and training frequency (*r* = 0.502, *p* < 0.001), years of practice (*r* = 0.437, *p* < 0.001), the CC scale (*r* = 0.237, *p* < 0.001), and MAAS (*r* = 0.161, *p* < 0.01)

**TABLE 3:** Correlation coefficients.

	Competence level
Training frequency	0.502**
Years of climbing	0.437**
CSES	0.237**
MAAS	0.161*
D1	0.082
D9	0.082
DFS TOT	0.081
RSES	0.069
D4	0.066
D6	0.056
TEIQue_Sc	0.049
D7	0.034
D3	0.028
D5	0.028
TEIQue_S	0.025
TEIQue Tot	0.013
TEIQue_W	0.005
D2	0.005
D8	−0.003
TEIQue_E	−0.084

**p ≤ 0.01,

*p ≤ 0.05.

### Structural equation model

**FIGURE 1:**
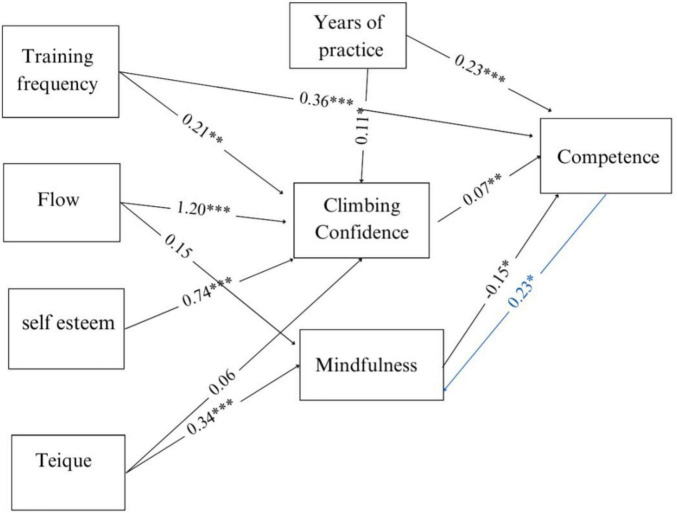
Structural Equation Model (SEM) of pychological influencing climbing competence. ***p ≤ 0.001, **p ≤ 0.01, *p ≤ 0.05.

A structural equation model (SEM) was conducted to test the hypotheses and observe the interaction of psychological factors, directly and indirectly influencing the competence of the climbers’ group. The model is represented in Figure 1. The goodness-of-fit statistics for the SEM model were all within acceptable ranges, with all indices indicating a good fit. Specifically, we evaluated several indices following established guidelines (Kline, 2015), with the following results: RMSEA = 0.08, CFI = 0.97, TLI = 0.90, and SRMR = 0.03. The Chi-square test was significant (χ^2^ = 19.74, df = 6; *p* < 0.003). According to widely accepted criteria for SEM model fit, RMSEA and TLI both reached the minimum threshold for an acceptable fit, although values closer to 0.95 are preferred for TLI (Schermelleh-Engel et al., 2003) and values below 0.08 are generally considered a better fit for RMSE (MacCallum et al., 1996). Moreover, both CFI and SRMR values (respectively, 0.97 and 0.03) suggested a strong model fit, since the commonly recommended threshold for CFI is 0.95 (or above) and for SRMR is equal or below 0.08 (Hu and Bentler, 1999). On the other hand, the Chi-square statistics resulted significant. However, this result is often found in larger samples and more complex models and given that all other indices suggest a good fit, the significant Chi-square does not necessarily indicate a misfit (Kline, 2015). Climbing confidence (CC), mindfulness (MAAS), years of practice, and training frequency were all significant predictors of competence (CC β = 0.07, MAAS β = −0.15, years of practice β = 0.23, training frequency β = 0.36). Unexpectedly, MAAS was found to be a negative predictor of competence. The CC scale is significantly and positively predicted by self-esteem (β = 0.74), flow (β = 1.20), years of practice (β = 0.11), and training frequency (β = 0.21). Emotional Intelligence (Teique) was also a significant predictor of mindfulness (β = 0.34). The influence of competence on mindfulness was also analyzed and, in this case, it was observed that competence is a significant positive predictor of mindfulness (β = 0.23). The levels of explained variance were significant for mindfulness, competence, and climbing confidence, respectively (R^2^ = 0.13, 0.39, 0.31).

### Power considerations: Retrospective design analysis

Although we did not conduct an *a priori* power analysis before data collection, we used a Retrospective Design Analysis (RDA) to evaluate the robustness of our findings *post hoc*, following the recommendations of Gelman and Carlin (2014). RDA is not a replacement for prospective power analysis, but it can offer insight into which observed effects are likely to be reliably detected and which may be prone to inferential risks. Based on our sample size of 365 participants and hypothesized small-to-medium effect sizes, we examined potential risk associated with low statistical power. The results showed that effects such as Climbing Confidence → Competence (β = 0.07) and Climbing Confidence → Mindfulness (β = 0.06) were below conventional thresholds for robust detection (power < 0.80). Therefore, these small effects may be susceptible to overestimation (Type M error) or misdirection (Type S error), and should be interpreted with caution. In contrast, larger effects such as Flow → Climbing Confidence (β = 1.20, p < 0.001) were associated with high statistical power, suggesting they are more robust and less likely to reflect Type M or S errors.

Overall, while our model appears adequately powered to detect medium-to-large effects (β≈ 0.3–1.20), but the smaller observed effects (β = 0.06–0.07) may be underpowered and require cautious interpretation. Future replication with larger samples may help clarify the stability of these associations and may help to validate the weaker effects.

This section is intended to enhance transparency and should not be considered a substitute for prospective planning in future research.

## Discussion

The aim of this exploratory study was to investigate the existing relationship between competence in sport climbing and psychological variables, considering training frequency and years of practice as independent variables. Our hypothesis that self-efficacy, self-esteem, mindfulness, emotional intelligence, and flow would influence competence was partially supported by the findings.

Correlation analysis revealed that competence levels were positively associated with training frequency, years of climbing experience, mindfulness, and domain-specific climbing confidence. To further examine the direction and natura of these relationships, we employed a structural equation model (SEM). Among the psychological dispositions, climbing confidence emerged as the strongest predictor of competence. This aligns with prior research emphasizing the importance of domain-specific self-efficacy in sport climbing performance (Mangan et al., 2024; Saul et al., 2019). Individuals with higher climbing confidence may be more likely to challenge themselves, attempt difficult routes, and build skill through repeated exposure.

Our findings further show that climbing confidence was positively predicted by years of practice, training frequency, general self-esteem, and the disposition to experience flow. Flow, in this context, refers to the optimal psychological state during which a climber becomes fully immersed in the climbing activity. Prior studies have shown that flow is often experienced during phases of route planning and execution (Boudreau et al., 2022), and may facilitate performance by enhancing focus, coordination, and the integration of motor schemas (Saul et al., 2019; Rucińska, 2021).

Interestingly, trait emotional intelligence (EI) was not a significant predictor of climbing confidence, suggesting that general emotional dispositions may not strongly translate to domain-specific performance perceptions. This is consistent with the findings of Garrido-Palomino and España-Romero (2019), who noted that while EI might differentiate among climbers at different skill levels, its predictive power may depend on the type of EI measured used (trait vs. ability-based). Future work may benefit from incorporating ability-based assessments to better capture the dynamic regulation of emotion in performance contexts.

We also found that EI was positively associated with mindfulness. This relationship suggests a potential indirect pathway by which emotional traits enhance sport climbing competence, likely through improved emotional awareness, attention regulation, and resilience. Dispositional mindfulness has been associated with adaptive emotional regulation and the ability to stay focused under pressure (Cerin and Barnett, 2011), both of which are essential when managing stressors such as fear of falling. Moreover, being persistent in pursuing long-term goals despite challenges reflects the psychological trait of grit, which has shown strong associations with sport performance (Duckworth et al., 2007) and climbing in particular (Ionel et al., 2023). These traits may contribute to athletes’ ability to confront emotionally demanding situations in climbing without resorting to avoidance or disengagement, as also observed by Steca et al. (2018) in their examination of emotional stability and self-regulation in athletic contexts.

A noteworthy finding from our model is the bidirectional relationship between mindfulness and climbing competence. Specifically, higher mindfulness levels negatively predicted competence, whereas higher competence positively predicted mindfulness. This may indicate that individuals with greater mindfulness dispositions are not necessarily advanced climbers, perhaps due to lifestyle or personality characteristics unrelated to climbing experience. Conversely, as climbers gain experience and confidence, their ability to maintain focused awareness during complex climbs may increase, reflecting a performance-enhanced development of mindfulness. These interpretations align with findings by Wheatley (2023), who observed that bouldering training, but not strength and conditioning, improved mindfulness among young adults.

This observation is also supported by research framed within the Embodied Cognition (EC) perspective, which emphasizes the feedback loop between sensorimotor engagement and cognitive development (Rucińska, 2021; Carius et al., 2020). Engaging in complex motor activities like sport climbing may enhance athletes’ bodily awareness and attentional control through repetitive, high-demand tasks.

First, the gender distribution in our sample was skewed toward males, limiting the generalizability of findings. This mirrors broader trends in sport climbing research (Boudreau et al., 2022; Diez-Fernández et al., 2023). As female participation grows (Sport e Salute, 2023), future research should strive for greater representation to avoid perpetuating gender bias in this emerging field.

Second, data collection occurred during the semi-lockdown period of the COVID-19 pandemic (November 2020–April 2021), which may have influenced participants’ training patterns, emotional states, and psychological responses. While most research during this period has focused on elite athletes, few studies address the effects of pandemic conditions on non-elite recreational sport participants (Carnevale Pellino et al., 2022).

Third, although we included a Retrospective Design Analysis (RDA), the lack of an *a priori* power analysis limits the strength of our conclusions, particularly regarding small effect sizes. RDA should be viewed as an exploratory tool rather than a substitute for prospective planning. Future studies should incorporate preregistered protocols and power estimations.

This study offers insights for training approaches that integrate both psychological and physical components. Enhancing domain-specific self-efficacy—climbing confidence—may be a key strategy for improving competence. Moreover, fostering mindfulness and the capacity for flow could further support climbers’ psychological resilience and focus. Coaches and sport psychologists might consider incorporating mindfulness techniques and flow-inducing strategies into training programs, particularly in preparation for complex route navigation and high-pressure scenarios.

Our findings highlight the multifaceted nature of sport climbing competence, shaped by a combination of experience, training frequency, and psychological dispositions. They also underscore the importance of theoretical precision and population specificity in recreational sport psychology research. Future studies should explore how motivational climates and social dynamics shape performance outcomes in sport climbing (Lacerda et al., 2021; Bortoli et al., 2011), contributing to a more nuanced understanding of this rapidly growing discipline.

## Data Availability

The original contributions presented in the study are included in the article/supplementary material, further inquiries can be directed to the corresponding author.
